# Sunitinib treatment does not improve blood supply but induces hypoxia in human melanoma xenografts

**DOI:** 10.1186/1471-2407-12-388

**Published:** 2012-09-04

**Authors:** Jon-Vidar Gaustad, Trude G Simonsen, Marit N Leinaas, Einar K Rofstad

**Affiliations:** 1 Group of Radiation Biology and Tumor Physiology, Department of Radiation Biology, Institute for Cancer Research, Oslo University Hospital, Montebello, Oslo, N-0310, Norway

**Keywords:** Antiangiogenic treatment, Sunitinib, Intravital microscopy, Vascular normalization, Tumor hypoxia

## Abstract

**Background:**

Antiangiogenic agents that disrupt the vascular endothelial growth factor pathway have been demonstrated to normalize tumor vasculature and improve tumor oxygenation in some studies and to induce hypoxia in others. The aim of this preclinical study was to investigate the effect of sunitinib treatment on the morphology and function of tumor vasculature and on tumor oxygenation.

**Methods:**

A-07-GFP and R-18-GFP human melanoma xenografts grown in dorsal window chambers were used as preclinical tumor models. Morphologic parameters of tumor vascular networks were assessed from high-resolution transillumination images, and tumor blood supply time was assessed from first-pass imaging movies recorded after a bolus of 155 kDa tetramethylrhodamine isothiocyanate-labeled dextran had been administered intravenously. Tumor hypoxia was assessed from immunohistochemical preparations of the imaged tissue by use of pimonidazole as a hypoxia marker.

**Results:**

Sunitinib treatment reduced vessel densities, increased vessel segment lengths, did not affect blood supply times, and increased hypoxic area fractions.

**Conclusion:**

Sunitinib treatment did not improve vascular function but induced hypoxia in A-07-GFP and R-18-GFP tumors.

## Background

Angiogenesis is necessary for tumors to grow beyond a certain size, determined by the diffusion limit of oxygen and other nutrients [1]. Several proteins that may stimulate or inhibit angiogenesis are produced and secreted by tumor cells, and the rate of tumor angiogenesis is regulated by the balance between these pro- and antiangiogenic factors [2]. While angiogenesis is tightly controlled in normal tissues and results in well-organized vasculature, malignant tumors show aberrant angiogenesis which results in an abnormal tumor vasculature. The vascular abnormalities include vessel wall abnormalities (i.e., incomplete or missing endothelial lining, interrupted or absent basement membrane, lack of pericytes and contractile vessel wall components), and architectural abnormalities (i.e., contour irregularities, elongated and tortuous vessels, limited arterial supply, loss of vessel hierarchy, existence of arterioveneous shunts, heterogeneous vessel distribution, and increased intervessel distances) [3-5]. The architectural abnormalities collectively increase the geometric resistance against blood flow [6,7]. The elevated geometric resistance and the low and heterogeneous vessel density cause unstable blood flow, and low and heterogeneous blood supply [3,7]. Consequently, the abnormal tumor vasculature plays a key role in the development of the hostile tumor microenvironment, which is characterized by hypoxia, glucose deprivation, low extracellular pH, and high interstitial fluid pressure (IFP) [4,8]. The hostile tumor microenvironment causes resistance to therapy [4] and may promote malignant progression and metastatic dissemination [9].

Several antiangiogenic drugs are being investigated, including endogenous inhibitors of angiogenesis [10,11], monoclonal antibodies against pro-angiogenic factors [12] or their receptors [13], and small molecule tyrosine kinase inhibitors which may target multiple pro-angiogenic receptors [14,15]. Antiangiogenic therapy may inhibit tumor growth significantly when used as a single treatment modality, but the therapeutic benefit may be even greater when used in combination with conventional treatment modalities such as ionizing radiation and chemotherapy [16]. The mechanisms underlying the enhanced antitumor effects of the combined treatments may differ among the antiangiogenic agents, and have not been determined conclusively.

It has been suggested that antiangiogenic treatment can normalize the tumor vasculature and the tumor microenvironment and hence sensitize tumors to conventional therapy [17]. Thus it has been shown that attenuation of proangiogenic signaling may prune immature blood vessels and remodel others, resulting in more efficient vascular networks. The normalized vascular networks are characterized by greater pericyte coverage, lower vessel permeability, lower vessel tortuousity, and lower vessel density [18,19]. Some studies report decreased vessel diameters [13,18], whereas others report increased vessel diameters after antiangiogenic treatment [14,20]. Collectively, the changes in vascular morphology are expected to reduce the geometric resistance against blood flow and hence improve vascular function in remaining vessels. In accordance with this, antiangiogenic treatment has been reported to enhance blood perfusion [21,22], improve tumor oxygenation [13,19,23], lower IFP, and increase the delivery of chemotherapeutic agents [18,22]. The normalization effect is transient, as the tumors can switch to other angiogenesis pathways and thus become resistant to antiangiogenic agents [24]. The duration of improved tumor oxygenation is also expected to be limited because the beneficial effects on vascular function may be balanced by severe vascular regression by prolonged exposure to antiangiogenic agents [17]. The optimal antitumor effect of combination therapy is thus expected if ionizing radiation or chemotherapy is administered within the time-window of normalization induced by the antiangiogenic agent [17]. However, the effect of antiangiogenic therapy on tumor oxygenation is debated, and other studies have reported enhanced hypoxic fractions after antiangiogenic treatment [25-27]. The effect on oxygenation may depend on tumor model, type and dose of the antiangiogenic agent, and may vary with time.

Sunitinib is a small molecule tyrosine kinase inhibitor which targets vascular endothelial growth factor receptors 1–3 (VEGFR-1, -2, and -3), platelet-derived growth factor receptors α-β (PDGFR-α and PDGFR-β), stem cell growth factor receptor (c-KIT), and fms-like tyrosine kinase receptor 3 (FLT 3), with potent antiangiogenic and antitumor activity [28]. The clinical efficacy of sunitinib has been demonstrated for patients with gastrointestinal stromal tumors (GIST) or metastatic renal cell carcinoma (RCC) [29,30]. However, studies investigating whether sunitinib treatment can normalize tumor vasculature are scarce, and whether sunitinib treatment can improve tumor oxygenation is currently unknown. Therefore, the aim of this study was to investigate the effect of sunitinib treatment on the morphology and function of tumor vasculature, and on tumor oxygenation. For this purpose human melanoma xenografts grown in dorsal window chambers were examined by intravital microscopy techniques, and were subjected to immunohistological examination. We report that sunitinib treatment significantly reduced vessel density, did not improve vascular function, and induced tumor hypoxia.

## Methods

### Mice

Adult (8–10 weeks of age) female BALB/c *nu/nu* mice were used as host animals for dorsal window chamber preparations. The mice were bred at our institute and maintained under specific pathogen-free conditions at constant temperature (24–26°C) and humidity (30–50%). After implantation of dorsal window chambers, the mice were kept at a temperature of 32°C and a humidity of 60-70%. The animal experiments were approved by the Norwegian National Animal Research Authority and were done according to the Interdisciplinary Principles and Guidelines for the Use of Animals in Research, Marketing, and Education (New York Academy of Sciences, New York, NY).

### Cells and multicellular spheroids

A-07 and R-18 human melanoma cells [31] were constitutively transfected with green fluorescence protein (GFP) by lipofection. The transfected cells (A-07-GFP and R-18-GFP) used in the present experiments were obtained from our frozen stock and grown as monolayers in RPMI 1640 (25 mM HEPES and L-glutamine) supplemented with 13% bovine calf serum, 250 μg/mL penicillin, 50 μg/mL streptomycin, and 700 μg/mL (A-07-GFP) or 2200 μg/mL (R-18-GFP) genetecin. Multicellular spheroids were produced by seeding approximately 10^6^ cells in 30 mL medium in plastic tissue culture flasks coated with a thin layer of 1% agar (Oxoid Limited, Hampshire, United Kingdom). The flasks were agitated (10 periods per minute) for 2 h using a tilting platform, and aggregates of approximately 50 μm in diameter were formed. The spheroids were then allowed to grow in coated culture flasks before implantation in window chambers (liquid-overlay culture technique) [32]. Cells and spheroid cultures were incubated at 37°C in a humidified atmosphere of 5% CO2 in air and subcultured twice a week.

### Anesthesia

Window chamber implantation and intravital microscopy examinations were carried out with anesthetized mice. Fentanyl citrate (Janssen Pharmaceutica, Beerse, Belgium), fluanisone (Janssen Pharmaceutica), and midazolam (Hoffmann-La Roche, Basel, Switzerland) were administered intraperitoneally in doses of 0.63 mg/kg, 20 mg/kg, and 10 mg/kg, respectively. After surgery, the mice were given a single injection of buprenorphine (Temgesic; Schering-Plough, Brussels, Belgium) intraperitoneally in a dose of 0.12 mg/kg to relieve pain.

### Window chamber preparations

Window chambers were implanted into the dorsal skin fold as described previously [33]. Briefly, the chamber consisted of two parallel frames, and after implantation, the frames sandwiched an extended double layer of skin. Before the chamber was implanted, a circular hole with a diameter of approximately 6.0 mm was made in one of the skin layers. A plastic window with a diameter of 6.0 mm was attached to the frame on the surgical side with a clip to provide visual access to the fascial side of the opposite skin layer. Tumors were initiated by implanting spheroids or tumor specimens with a diameter of 200 to 400 μm onto the exposed skin layer.

### Sunitinib treatment

Sunitinb L-malate (LC Laboratories, Woburn, MA, USA) was dissolved in hydrochloric acid (1.0 molar ratio of sunitinib), polysorbate 80 (0.5%; Sigma-Aldrich, Schnelldorf, Germany), polyethylene Glycol 300 (10%; Sigma-Aldrich, Schnelldorf, Germany), sodium hydroxide (to adjust pH to 3.5), and sterile water. Mice were treated with 20 or 40 mg/kg/day sunitinib or vehicle for 4 or 8 days, by oral administration.

### Intravital microscopy

Intravital microscopy was performed before initiation of sunitinib treatment (day 0), and 1, 2, and 4 days after the start of treatment (short-term treatment), or before, and 2, 4, 6 and 8 days after the start of treatment (prolonged treatment). The mice were kept in a specially constructed holder that fixed the window chamber to the microscope stage during intravital microscopy. The body core temperature was kept at 37 to 38°C by using a hot-air generator. Imaging was performed by using an inverted fluorescence microscope equipped with filters for green and red light (IX-71; Olympus, Munich, Germany), a black and white CCD camera (C9300-024; Hamamatsu Photonics, Hamamatsu, Japan), and appropriate image acquisition software (Wasabi; Hamamatsu Photonics). Tumor vasculature was visualized by using transillumination and filters for green light, and tumor vascular networks were mapped by recording 1-4 single frames with a × 4 objective lens resulting in a field of view of 3.80 × 3.80 mm^2^ and a pixel size of 3.7 × 3.7 μm^2^. To study the function of tumor vasculature, first-pass imaging movies were recorded after a 0.2 mL bolus of 50 mg/mL tetramethylrhodamine isothiocyanate-labeled dextran (Sigma-Aldrich, Schnelldorf, Germany) with a molecular weight of 155 kDa was injected into the lateral tail vein. First-pass imaging movies were recorded at a frame rate of 22.3 frames per second by using a × 2 objective lens, resulting in a time resolution of 44.8 ms, a field of view of 5.97 × 5.97 mm^2^, and a pixel size of 7.5 × 7.5 μm^2^. All recordings were stored and analyzed offline. Tumor size (i.e., tumor area) was calculated from the number of pixels showing GFP fluorescence.

### Analysis of vascular morphology

**Figure 1:**
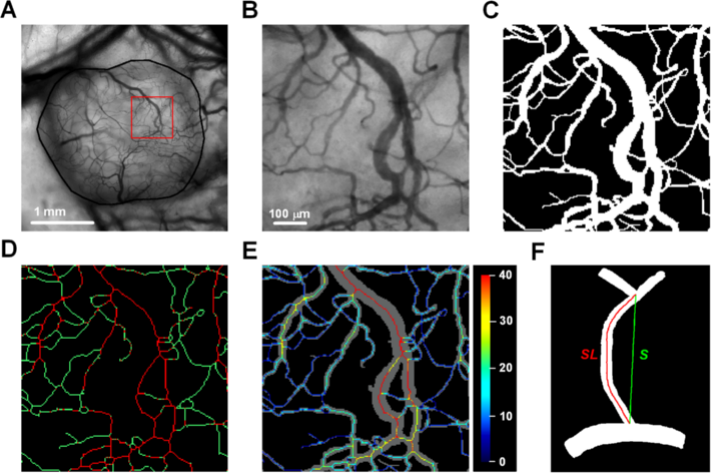
**Analysis of vascular morphology. A**, intravital microscopy image of a representative untreated R-18-GFP tumor. Tumor area is delineated by a solid black line. **B**, intravital microscopy image of the region highlighted in **A**. **C,** vascular mask. **D**, skeleton of the vascular mask. Vessels with diameter >15 μm are shown in red and vessels with diameter < 15 μm are shown in green. **E**, color-coded vessel diameter superimposed on the vascular mask. Color bar, vessel diameter scale in μm. Figures **C-E** refer to the tumor region shown in **B**. **F**, sketch of vessel segment. Vessel segment length (*SL*; red) was defined as the distance between branching points along the vessel, whereas vessel tortuosity (*T*) was defined as *T = (SL – S)· 100*% */ SL*, where *SL* represents the vessel segment length and *S* represents the shortest distance between the branching points (*S*; green).

Two-dimensional projected vascular masks were produced manually from transillumination images recorded with the × 4 objective lens. Interstitial distance (i.e., the distance from a tumor pixel outside the vascular mask to the nearest pixel within the vascular mask) and vessel diameter were computed from the vascular masks [34]. Vessel density (i.e., total vessel length per mm^2^ tumor area) was calculated from skeletons of vascular masks. Vessel density of small or large vessels was calculated from skeletons of vascular masks only showing vessels with a diameter smaller or larger than 15 μm. The analysis of vascular morphology is illustrated for a representative tumor in Figure 1. This figure shows high-resolution transillumination images (Figure 1A-B), the vascular mask (Figure 1C), the skeleton of the vascular mask (Figure 1D), and color-coded vessel diameter superimposed on the vascular mask (Figure 1E). Vessel segment length and vessel tortuosity were calculated from ~50 randomly selected vessel segments. Vessel tortuosity (*T*) was defined as *T = (SL – S)· 100*% */ SL*, where *SL* represents the segment length (i.e., the distance between the branching points along the vessel) and *S* represents the shortest distance between the branching points (i.e., the distance between the branching points along a straight line), as illustrated in Figure 1F. Change in vessel diameter was assessed by manually measuring the diameter of the same vessel segments on subsequent days.

### Analysis of vascular function

Two-dimensional projected vascular masks were produced from the movies recorded with the × 2 objective lens as described previously [34]. Blood supply time (BST) images were produced by assigning a BST value to each pixel of the vascular masks. The BST of a pixel was defined as the time difference between the frame showing maximum fluorescence intensity in the pixel and the frame showing maximum fluorescence intensity in the main tumor supplying artery, as described in detail previously [35].

### Immunohistochemical detection of tumor hypoxia, microvessels, vascular basement membrane, and pericytes

The tumors were resected immediately after the last intravital microscopy examinations and fixed in phosphate-buffered 4% paraformaldehyde. Pimonidazole [1-[(2-hydroxy-3-piperidinyl)-propyl]-2-nitroimidazole], administered as described previously [36], was used as hypoxia marker, CD31 was used as marker for endothelial cells, collagen IV was used as marker for vascular basement membrane, and α-smooth muscle actin (α-SMA) was used as marker for pericytes. Immunohistochemistry was done by using a peroxidase-based indirect staining method [36]. An anti-pimonidazole rabbit polyclonal antibody (gift from Prof. J.A. Raleigh, Department of Radiation Oncology, University of North Carolina School of Medicine, Chapel Hill, NC), an anti-CD31 rabbit polyclonal antibody (Abcam, Cambridge, United Kingdom), an anti-collagen IV rabbit polyclonal antibody (Abcam), or an anti-α-SMA rabbit polyclonal antibody (Abcam) was used as primary antibody. Diaminobenzidine was used as chromogen, and hematoxylin was used for counterstaining. Hypoxic area fractions were determined by image analysis. Number of microvessel profiles per mm^2^ of tumor tissue (#/mm^2^) was scored manually and used as parameter for microvascular density.

### Statistical analysis

Statistical comparisons of data were carried out by the Student’s t test when the data complied with the conditions of normality and equal variance. Under other conditions, comparisons were done by nonparametric analysis using the Mann-Whitney rank sum test. The Kolmogorov-Smirnov method was used to test for normality, and the Levene’s test was used to test for equal variance. Probability values of *P* < 0.05, determined from two-sided tests, were considered significant. The statistical analysis was performed by using the SigmaStat statistical software (SPSS Science, Chicago, IL, USA).

## Results

### Short-term sunitinib treatment did not affect tumor growth

**Figure 2:**
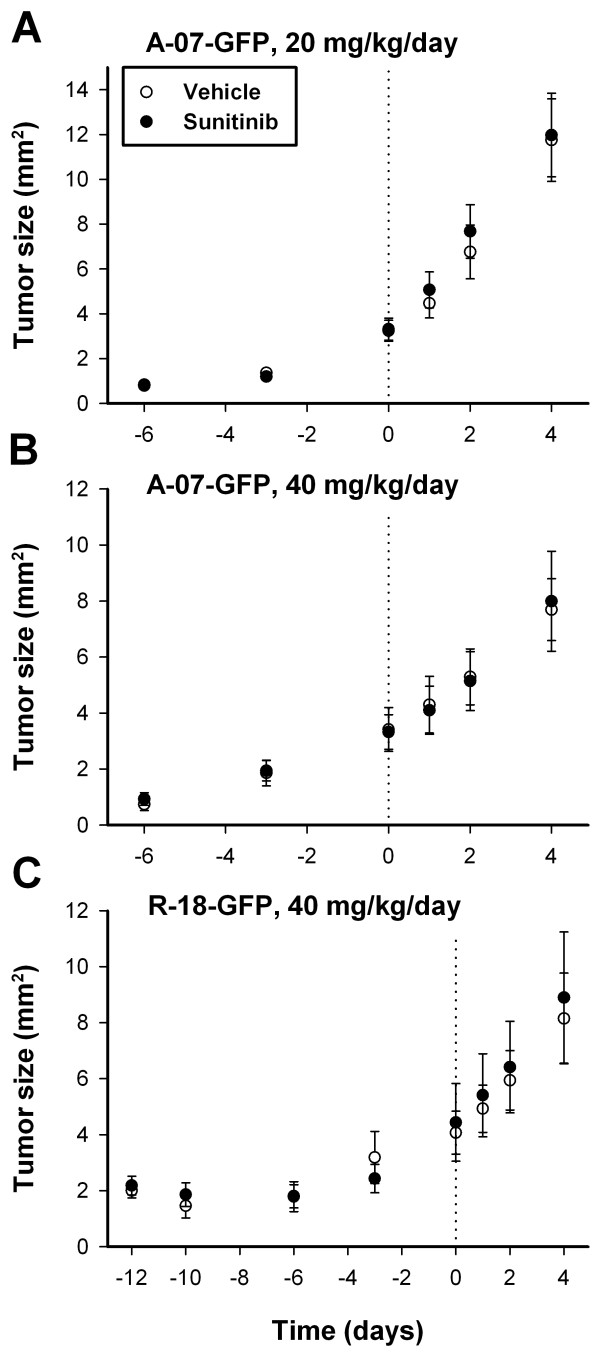
**Short-term sunitinib treatment did not affect tumor growth.** Tumor size versus time for untreated and sunitinib-treated A-07-GFP **(A-B)** and R-18-GFP tumors **(C)**. Points, means of 6-10 tumors; bars, SEM. Dotted line indicates time of treatment start. Tumors were initiated 6 (A-07-GFP; **A-B**) or 12 days (R-18-GFP; **C**) before start of treatment.

**Figure 3:**
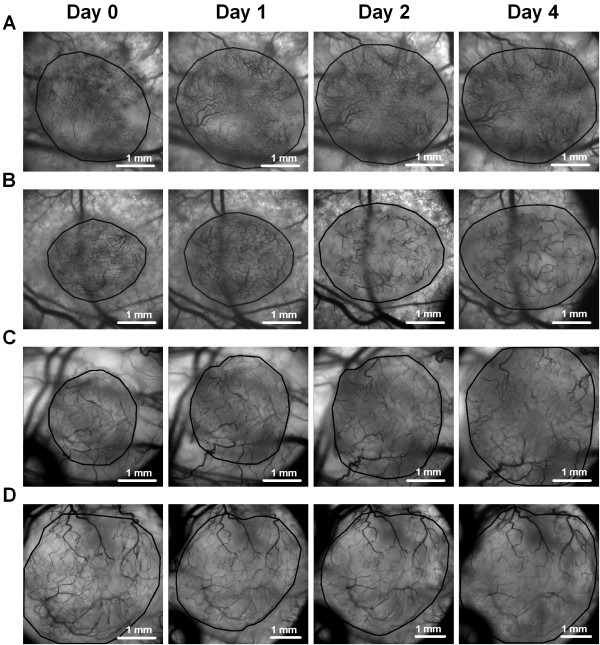
**Images of tumor vascular networks.** Representative intravital microscopy images from day 0, 1, 2, and 4 of an untreated A-07-GFP tumor **(A)**, an A-07-GFP tumor treated with 40 mg/kg/day sunitinib **(B)**, an untreated R-18-GFP tumor **(C)**, and an R-18-GFP tumor treated with 40 mg/kg/day sunitinib **(D)**. Tumor area is delineated by a solid black line.

Mice were divided in groups with matched tumor size to receive sunitinib treatment or no treatment (vehicle). Sunitinib treatment was started 6 (A-07-GFP) or 12 days (R-18-GFP) after tumor initiation. At these time points, defined as day 0, A-07-GFP and R-18-GFP tumors were of similar size (Figure 2), and had developed vascular networks (Figure 3). During the short treatment period, sunitinib-treated tumors did not differ from untreated tumors in size regardless of whether A-07-GFP tumors were treated with 20 mg/kg/day sunitinib (*P* > 0.05; Figure 2A), A-07-GFP tumors were treated with 40 mg/kg/day sunitinib (*P* > 0.05; Figure 2B), or R-18-GFP tumors were treated with 40 mg/kg/day sunitinib (*P* > 0.05; Figure 2C).

### Sunitinib treatment reduced vessel density

**Figure 4:**
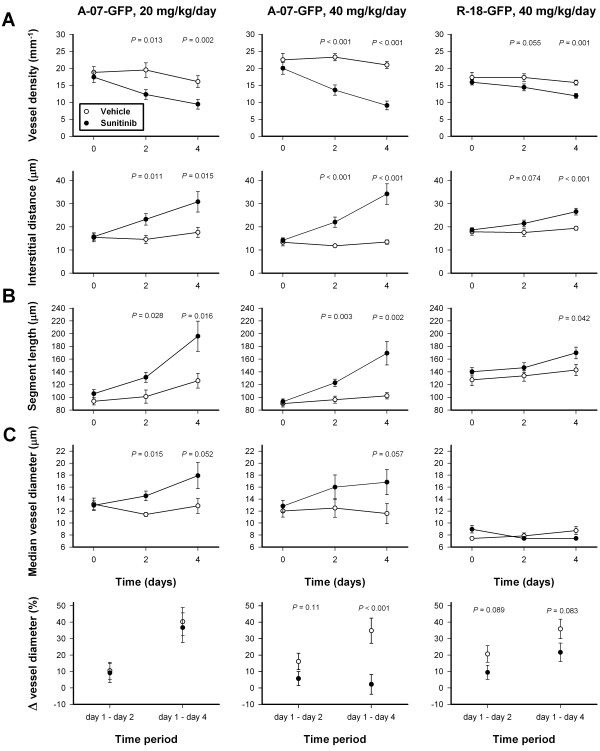
**Sunitinib treatment affected vascular morphology.** Vessel density, and median interstitial distance versus time **(A)**, median vessel segment length versus time **(B)**, median vessel diameter versus time, and change in vessel diameter versus time interval **(C)** for untreated and sunitinib-treated A-07-GFP and R-18-GFP tumors. *P*-values are indicated in the panels where statistical tests revealed significant or borderline significant differences between untreated and sunitinib-treated tumors. Points, means of 6-9 tumors; bars, SEM.

To investigate effects of sunitinib treatment on vascular morphology, mice treated with sunitinib or vehicle were submitted to intravital microscopy. Sunitinib treatment reduced vessel density in both A-07-GFP and R-18-GFP tumors. This is shown qualitatively in Figure 3 which shows representative intravital microscopy images of an untreated A-07-GFP tumor (Figure 3A), an A-07-GFP tumor treated with sunitinib (Figure 3B), an untreated R-18-GFP tumor (Figure 3C), and an R-18-GFP tumor treated with sunitinib (Figure 3D). To quantify these qualitative observations, vascular masks were produced and vessel densities and interstitial distances were calculated. Sunitinib-treated tumors had significantly lower vessel densities and significantly higher interstitial distances than untreated tumors (Figure 4A). The sunitinib-induced reduction in vessel densities was more pronounced for A-07-GFP tumors than for R-18-GFP tumors. For sunitinib-treated A-07-GFP-tumors, the density of small vessels decreased more than the density of large vessels (Table 1), implying that sunitinib treatment selectively pruned small vessels. For sunitinib-treated R-18-GFP tumors, the densities of small and large vessels were similarly reduced.

**Table 1:** Reductions in vessel density after short-term sunitinib treatment

	**All vessels**	**Small vessels**	**Large vessels**
**A-07-GFP 20 mg/kg/day**	1.71	2.11	1.40
**A-07-GFP 40 mg/kg/day**	2.31	2.99	1.75
**R-18-GFP 40 mg/kg/day**	1.33	1.31	1.37

Data refer to fold reduction in vessel density in sunitinib-treated tumors compared with untreated tumors after 4 days of treatment (i.e., vessel density in untreated tumors / vessel density in sunitinib-treated tumors). Vessel densities refer to the vessel density of all tumor vessels, only small tumor vessels (vessels with diameter < 15 μm), or only large tumor vessels (vessels with diameter > 15 μm).

### Sunitinib treatment increased vessel segment length, but did not affect vessel tortuosity

To investigate the effect of sunitinib treatment on individual vessels, vessel segment lengths and vessel tortuosities were quantified. Sunitinib-treated tumors had significantly longer segment lengths than untreated tumors (Figure 4B), whereas sunitinib treatment did not affect vessel tortuosities (*P* > 0.05; data not shown). The sunitinib-induced effect on segment lengths was more pronounced for A-07-GFP tumors than for R-18-GFP tumors.

### Sunitinib treatment increased median vessel diameter but inhibited vessel diameter increase in remaining vessels

Sunitinib-treated A-07-GFP tumors showed higher median vessel diameters than untreated A-07-GFP tumors, whereas sunitinib-treated R-18-GFP tumors did not differ from untreated R-18-GFP tumors in median vessel diameter (Figure 4C). To investigate changes in vessel diameters of remaining vessels, the diameter was measured in the same vessels on subsequent days. Vessels in untreateted tumors showed an increase in vessel diameter from day 1 to day 2, and from day 1 to day 4. Vessels in A-07-GFP tumors treated with 20 mg/kg/day sunitinib showed similar changes in vessel diameter, whereas A-07-GFP and R-18-GFP tumors treated with 40 mg/kg/day sunitinib showed lower increases in vessel diameters than untreated tumors (Figure 4C). Consequently, the increase in median vessel diameter did not reflect increases in the vessel diameters of remaining vessels, but rather a selective pruning of small vessels.

### Immunohistochemical investigations showed that sunitinib treatment did not affect vascular basement membrane and pericyte-coverage and confirmed that sunitinib treatment reduced vessel density

**Figure 5:**
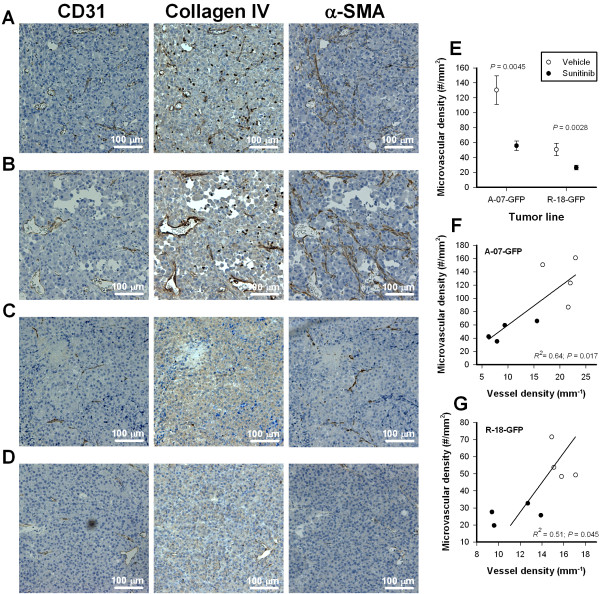
**Immunohistochemical detection of microvessels, vascular basement membrane, and pericytes. A-D**, representative immunohistochemical preparations stained with anti-CD31 antibody, anti-collagen IV antibody, or anti-α-smooth muscle actin (α-SMA) antibody to visualize endothelial cells, vascular basement membrane, and pericytes. The images refer to an untreated A-07-GFP tumor (**A**), an A-07-GFP tumor treated with 40 mg/kg/day sunitinib (**B**), an untreated R-18-GFP tumor (**C**), and an R-18-GFP tumor treated with 40 mg/kg/day sunitinib (**D**). **E**, microvascular density in untreated and sunitinib-treated A-07-GFP and R-18-GFP tumors. Points, means of 4 tumors; bars, SEM. **F-G**, microvascular density assessed by immunohistochemistry versus vessel density assessed by intravital microscopy in A-07-GFP (**F**) and R-18-GFP (**G**) tumors. Points, individual tumors; curves, curves fitted to the data by linear regression analysis.

Histological sections of A-07-GFP and R-18-GFP window chamber tumors were stained for CD31, collagen IV, or α-SMA to visualize endothelial cells, vascular basement membrane, and pericytes. Vessels in A-07-GFP tumors showed vascular basement membrane and were covered with pericytes (Figure 5A-B). Long bands with positive α-SMA staining originating in CD31-positive vessel walls were observed in A-07-GFP tumors, revealing large networks of pericytes in these tumors. In contrast, most vessels in R-18-GFP tumors did not show vascular basement membrane, and pericytes were only found adjacent to microvessels (Figure 5C-D). Sunitinib-treated tumors did not differ from untreated tumors in vascular basement membrane or pericyte-coverage and, consequently, the differences in vessel maturation between A-07-GFP and R-18-GFP tumors were observed regardless of whether untreated or sunitinib-treated tumors were considered. Immunohistochemical preparations stained for CD31 confirmed that sunitinib treatment significantly reduced vessel density (Figure 5E). Moreover, significant correlations were found between microvascular density assessed by immunohistochemistry and vessel density assessed by intravital microscopy in both A-07-GFP and R-18-GFP tumors (Figure 5F-G).

### Sunitinib treatment did not affect BST

**Figure 6:**
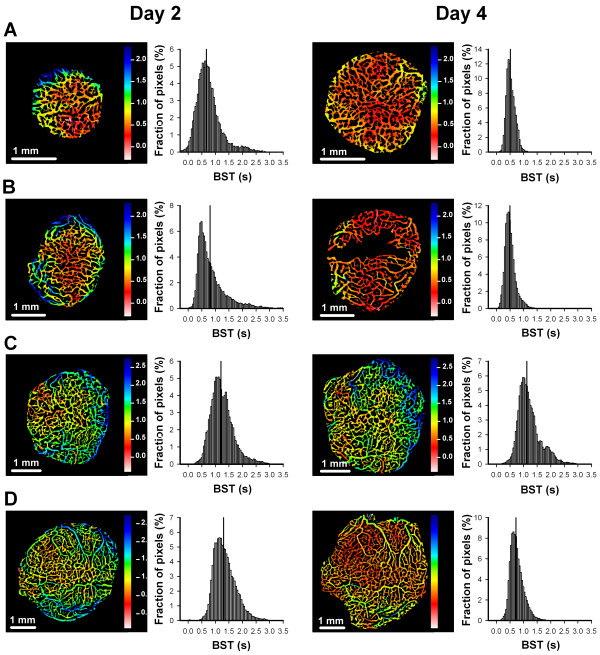
**BST images and frequency distributions.** Representative blood supply time (BST) images and the corresponding BST frequency distributions from day 2 and 4 of an untreated A-07-GFP tumor **(A)**, an A-07-GFP tumor treated with 40 mg/kg/day sunitinib **(B)**, an untreated R-18-GFP tumor **(C)**, and an R-18-GFP tumor treated with 40 mg/kg/day sunitinib **(D)**. Color bars, BST scale in seconds; vertical lines, median BST.

**Figure 7:**
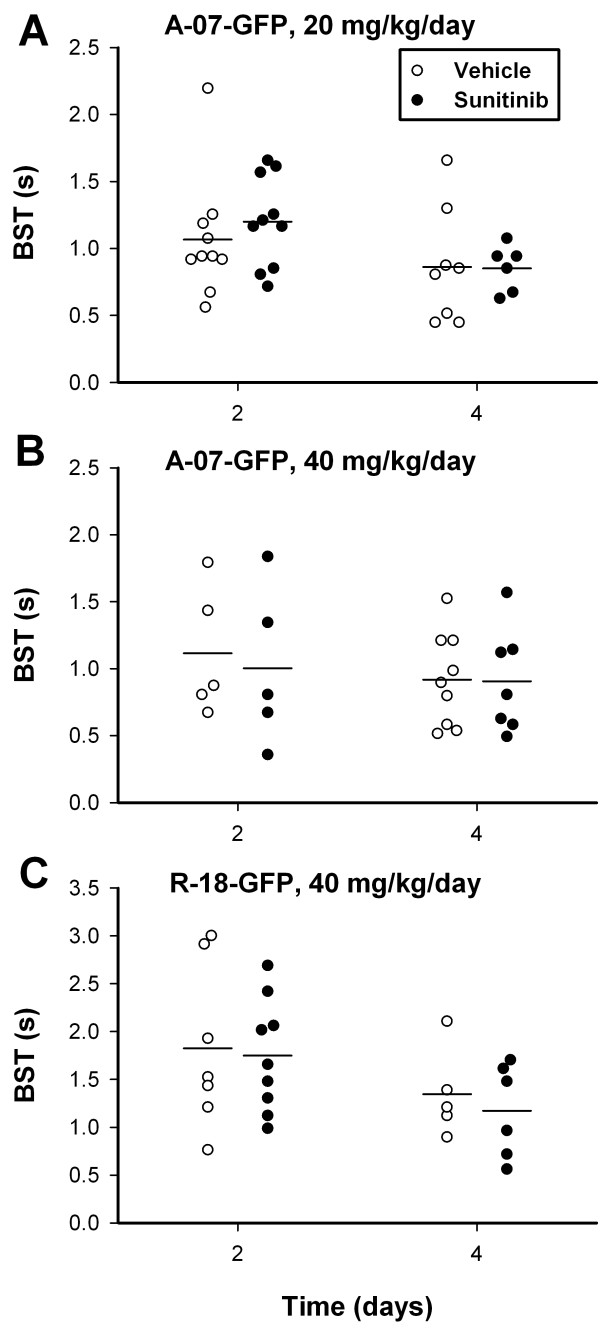
**Sunitinib treatment did not affect BST.** Median blood supply time (BST) at day 2 and 4 for untreated and sunitinib-treated A-07-GFP and R-18-GFP tumors. Points, individual tumors; horizontal lines, mean BST.

To investigate the effect of sunitinib treatment on the function of tumor vasculature, first-pass imaging movies were recorded, and BST images and BST frequency distributions were produced. BST was not affected by sunitinib treatment. This is shown qualitatively in Figure 6, which shows representative BST images and the corresponding BST frequency distributions from day 2 and 4 of an untreated A-07-GFP tumor (Figure 6A), an A-07-GFP tumor treated with sunitinib (Figure 6B), an untreated R-18-GFP tumor (Figure 6), and an R-18-GFP tumor treated with sunitinib (Figure 6D). The black region in the sunitinib-treated A-07-GFP tumor (Figure 6B) reflects an avascular region which contains hypoxic tumor tissue. Figure 7 shows BST for all tumors included in the short-term treatment experiments, illustrating that untreated and sunitinib-treated tumors did not differ in BST on either day 2 or 4, regardless of whether A-07-GFP tumors were treated with 20 mg/kg/day sunitinib (*P* > 0.05; Figure 7A), A-07-GFP tumors were treated with 40 mg/kg/day sunitinib (*P* > 0.05; Figure 7B), or R-18-GFP tumors were treated with 40 mg/kg/day sunitinib (*P* > 0.05; Figure 7C).

### Sunitinib treatment induced hypoxia

**Figure 8:**
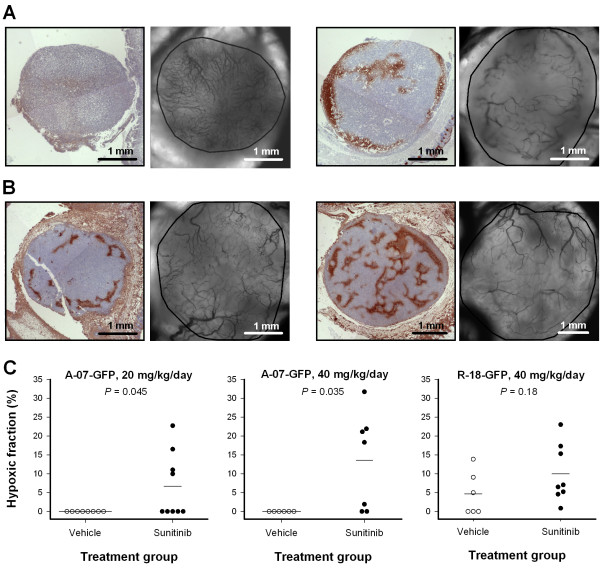
**Sunitinib treatment induced hypoxia. A-B**, representative immunohistochemical preparations stained for pimonidazole to visualize hypoxia and intravital microscopy images of an untreated A-07-GFP tumor (**A**, left), an A-07-GFP tumor treated with 40 mg/kg/day sunitinib (**A**, right), an untreated R-18-GFP tumor (**B**, left), and an R-18-GFP tumor treated with 40 mg/kg/day sunitinib (**B**, right). Tumor area is delineated by a solid black line in intravital microscopy images. **C**, hypoxic area fraction for untreated and sunitinib-treated A-07-GFP and R-18-GFP tumors. Points, individual tumors; horizontal lines, mean hypoxic area fraction.

To investigate the effect of sunitinib treatment on tumor hypoxia, tumors were resected and submitted to histological examination immediately after the last intravital microscopy imaging session. Untreated A-07-GFP tumors did not show hypoxic regions whereas sunitinib-treated A-07-GFP tumors showed multiple hypoxic regions (Figure 8A). The hypoxic regions co-localized with avascular regions or regions with very low vessel density, and were found in both central and peripherial parts of sunitinib-treated A-07-GFP tumors (Figure 8A; right). 3 out of 6 untreated R-18-GFP tumors and 7 out of 8 sunitinib-treated R-18-GFP tumors showed scattered hypoxic regions (Figure 8B). R-18-GFP tumors did not show avascular regions, and the hypoxic regions reflected low overall vessel densities. The hypoxic area fractions were significantly higher in sunitinib-treated A-07-GFP tumors than in untreated A-07-GFP tumors, and a non-significant trend towards higher hypoxic area fractions in sunitinib-treated R-18-GFP tumors compared to untreated R-18-GFP tumors was observed (Figure 8C).

### Prolonged sunitinib treatment reduced tumor growth

**Figure 9:**
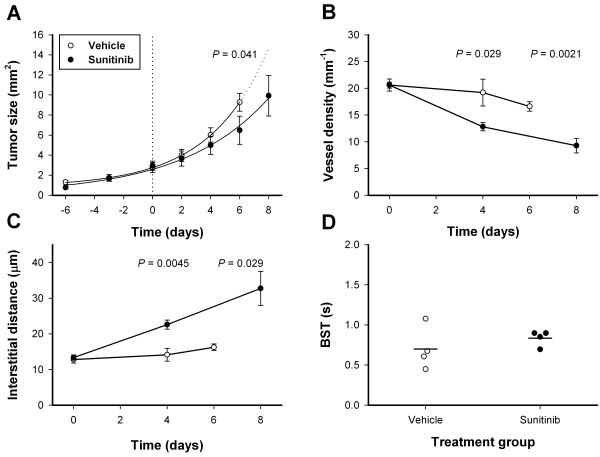
**Prolonged sunitinib treatment reduced tumor growth. A**, tumor size versus time for untreated and sunitinib-treated A-07-GFP tumors. Points, means of 4 tumors; bars, SEM. Curves, curves fitted to the data by regression analysis. Dotted vertical line indicates time of treatment start. Tumors were initiated 6 days before start of treatment. **B-C**, vessel density **(B)**, and interstitial distance **(C)** versus time for untreated and sunitinib-treated A-07-GFP tumors. Points, means of 4 tumors; bars, SEM. **D**, median blood supply time (BST) for untreated and sunitinib-treated A-07-GFP tumors. Points, individual tumors; horizontal lines, mean BST. *P*-values are indicated in the panels where statistical tests revealed significant differences between untreated and sunitinib-treated tumors **(A-D).**

In a separate experiment, A-07-GFP tumors were treated with 40 mg/kg/day sunitinib or vehicle for 8 days. By day 6, untreated tumors grew close to the window chamber boundaries, and mice bearing untreated tumors were sacrificed to avoid growth restriction by the chamber preparations. Sunitinib-treated tumors were significantly smaller (*P* = 0.041; Figure 9A), and were allowed to grow for two more days. The prolonged sunitinib treatment further reduced vessel densities, and increased interstitial distances (Figure 9B-C), but did not affect BST (Figure 9D). Our experimental model did not allow evaluation of longer treatment periods. The treatment was started immediately after tumors were vascularized, and the experiments were ended before the tumors outgrew the window chambers. Sunitinib treatment completely inhibited vascularization if the treatment was started before tumors were vascularized (data not shown).

## Discussion

In the present work, A-07-GFP and R-18-GFP melanoma xenografts grown in dorsal window chambers were used as preclinical models. It has previously been shown that intradermal A-07 and R-18 xenografts retain several characteristic features of the original patient tumors, including histological appearance, angiogenic potential, and vessel density [31]. Moreover, intradermal A-07 and R-18 xenografts have been shown to differ substantially in angiogenic potential, vessel density, growth rate, and oxygenation status [31,36,37]. These differences were maintained when A-07-GFP and R-18-GFP tumors were grown in dorsal window chambers despite the transfection with GFP, the confinement of tumor growth by the chamber preparations, the small size of the tumors, and the elevated temperature during tumor growth. Consequently, A-07-GFP and R-18-GFP tumors grown in dorsal window chambers should be appropriate models for investigating the effect of sunitinib treatment on tumor vasculature and oxygenation.

Tumors were treated with two different doses of sunitinib in the current study. Both sunitinib doses have been shown to result in sufficient plasma concentrations in athymic mice to inhibit VEGFR and PDGFR phosphorylation in xenografts of human melanoma, human glioma, and human colon carcinoma [38]. Lower sunitinib doses have been shown to result in insufficient plasma concentrations and no inhibition of VEGFR and PDGFR phosphorylation in the same xenograft models [38]. Moreover, the higher sunitinib dose (40 mg/kg/day) has been shown to reduce vessel density and improve vascular function in human glioma xenografts [14]. The two sunitinib doses should therefore be well suited to evaluate the effect of sunitinib treatment on tumor vasculature and tumor oxygenation.

The morphology of tumor vasculature was assessed by mapping tumor vascular networks with high-resolution transillumination images and filters for green light. In these images only vessels with erythrocytes can be seen and, consequently, the morphological analysis was based on vessels with erythrocytes as opposed to dysfunctional vessels with plasma only. The function of tumor vasculature was assessed by using a novel first-pass imaging method which involves recording movies of the dynamic distribution of a fluorescent vascular tracer after an intravenous bolus injection [33,35,39]. From the recorded first-pass imaging movies, BST images and corresponding BST frequency distributions were produced. We have previously shown that the BST-assay is highly reproducible, sufficiently sensitive to detect gradients in BST along vessel segments, and sufficiently sensitive to indentify the majority of tumor vessels [33,35,39].

It has previously been shown that A-07 and R-18 cells express and secrete VEGF-A and interleukin-8 (IL-8), and that the angiogenic activity can be significantly reduced by inhibiting VEGF-A in both xenograft lines [40]. The secretion rate of VEGF-A and IL-8 has been shown to be higher for A-07 cells than for R-18 cells and, in addition, A-07 cells have been shown to express and secrete basic fibroblast growth factor (bFGF) whereas R-18 cells do not secrete this factor [40]. In the present work, we show that sunitinib treatment significantly reduced vessel densities in both A-07-GFP and R-18-GFP tumors. The sunitinib-induced antiangiogenic effects were more pronounced for A-07-GFP tumors than for R-18-GFP tumors, and these differences probably reflected differences in the angiogenic profiles. Treatment-induced reductions in vessel densities are expected to reduce tumor growth. In the present study, prolonged sunitinib treatment significantly reduced tumor growth, whereas short-term sunitinib treatment did not affect tumor size. This observation illustrates that effects on tumor size generally are delayed compared to the effects on tumor vasculature after antiangiogenic treatment [41].

Sunitinib treatment did not affect BST and, consequently, improved vascular function was not observed in the current study. Narrow, elongated, and tortuous tumor vessels are expected to elevate the geometric resistance to blood flow, and a potential increase in vessel diameter, decrease in vessel segment length, or reduction in vessel tortuosity is expected to enhance tumor blood flow [6,7]. In the present study, we observed increased vessel segment lengths and unchanged vessel tortuosities after sunitinib treatment, and the remaining vessels in sunitinib-treated tumors showed similar or smaller increases in vessel diameter than vessels in untreated tumors. The effects on vascular morphology are thus in accordance with the observation that sunitinib treatment did not improve vascular function. Reduction in vessel density combined with no improvement of vascular function is expected to impair oxygen supply. In accordance with this, sunitinib treatment induced hypoxia in A-07-GFP and R-18-GFP tumors. Interestingly, A-07-GFP tumors differed substantially from R-18-GFP tumors in vessel maturation. Our study thus illustrates that sunitinib treatment fails to improve vascular function in melanoma xenografts with both high and low degrees of vessel maturation.

Sunitinib-induced improvement of vascular function has been reported in a preclinical study of human glioma xenografts [14]. In that study, increased red blood cell velocities were observed 2, 4, and 6 days after the start of sunitinib treatment. The time points where improved vascular function was observed in that study thus correspond well to the time points where BST was assessed in our study (2, 4, and 8 days after the start of sunitinib treatment). Consequently, the lack of improved vascular function observed in our study was unlikely to be due to inadequate observation time points.

Treatment with anti-VEGF-A or anti-VEGFR-2 antibody has improved vascular function and tumor oxygenation in some preclinical models [13,19,23]. The same antibodies have failed to improve vascular function and increased hypoxic fractions or have not affected tumor oxygenation in other preclinical models [19,25]. Similarly, sunitinib-induced blockade of VEGFR and PDGFR has been reported to improve vascular function in human glioma xenografts [14], whereas the current study shows that sunitinib treatment does not improve vascular function in human melanoma xenografts. Consequently, whether antiangiogenic treatment improves vascular function, does not reflect whether the antiangiogenic agent blocks PDGFR in addition to VEGFR, but more likely reflects differences in tumor models.

In addition to improve tumor blood supply and oxygenation, antiangiogenic agents have also been reported to reduce vessel permeability and lower tumor IFP in preclinical models. These effects have collectively been referred to as vascular normalization [17,18]. Moreover, increased hypoxic fractions, reduced vessel permeability, and lowered tumor IFP have been observed simultaneously after antiangiogenic treatment [27]. This observation suggests that improved tumor oxygenation, normalized vessel permeability, and normalized tumor IFP may not necessarily occur in parallel temporal windows [27]. Consequently, although sunitinib treatment did not improve blood supply and oxygenation in A-07-GFP and R-18-GFP tumors, we cannot rule out the possibility that sunitinib treatment may normalize vessel permeability and tumor IFP in these tumor models.

Sunitinib has been shown to prolong progression-free and overall survival in patients with imatinib-refractory GIST and metastatic RCC in clinical phase III trials, and has been approved by the US Food and Drug Administration for these indications [29,30]. However, the tumors eventually become unresponsive to sunitinib, and the benefits in progression-free and overall survival are measured in months. Treatment regimes that combine sunitinib with ionizing radiation, different chemotherapeutic agents, or other antiangiogenic agents may enhance and prolong the effects of sunitinib, and clinical studies that evaluate such combinations are ongoing [42].

The current study and previously reported preclinical studies suggest that antiangiogenic treatment improves vascular function and tumor oxygenation in some clinical tumors, and induces hypoxia in others. Neoadjuvant antiangiogenic therapy may enhance the effect of ionizing radiation or chemotherapy in clinical tumors where antiangiogenic treatment improves vascular function. The feasibility of this strategy has been demonstrated in preclinical studies where maximal antitumor effect of ionizing radiation was achieved when tumors were irradiated within the time period when tumor oxygenation was improved by antiangiogenic treatment [13,23]. On the other hand, in clinical tumors where antiangiogenic treatment induces hypoxia, neoadjuvant antiangiogenic therapy is expected to reduce the effect of ionizing radiation or chemotherapy [4,8]. Consequently, antiangiogenic agents should not be considered as neoadjuvant therapy in combination with radiation or chemotherapy in such tumors. Whether it is possible to predict if antiangiogenic treatment can improve vascular function in a specific tumor is currently unknown. The effect of antiangiogenic treatment on tumor vasculature and on tumor oxygenation should thus be monitored closely if antiangiogenic treatment is considered as neoadjuvant therapy.

## Conclusions

Sunitinib treatment reduced vessel density but did not improve vascular function in A-07-GFP and R-18-GFP human melanoma xenografts. The sunitinib treatment increased hypoxic fractions and, consequently, sunitinib used as neoadjuvant therapy may reduce the effect of ionizing radiation or chemotherapy in clinical tumors similar to the A-07-GFP and R-18-GFP human melanoma xenografts.

## Abbreviations

α-SMA: α-smooth muscle actin; bFGF: basic fibroblast growth factor; BST: blood supply time; c-KIT: stem cell growth factor receptor; FLT 3: fms-like tyrosine kinase receptor 3; GFP: green fluorescent protein; GIST: gastrointestinal stromal tumor; IFP: interstitial fluid pressure; IL-8: interleukin-8; PDGFR: platelet-derived growth factor receptor; RCC: renal cell carcinoma; VEGFR: vascular endothelial growth factor receptor.

## Competing interests

The authors declare that they have no competing interests.

## Authors’ contributions

JVG, TGS, and EKR conceived and designed the study. JVG, TGS, and MNL performed the experiments. JVG, TGS, and EKR analyzed and interpreted the data. JVG and EKR wrote the manuscript. All authors read and approved the final manuscript.

## Pre-publication history

The pre-publication history for this paper can be accessed here:

http://www.biomedcentral.com/1471-2407/12/388/prepub
